# The Marine Boundary Layer Height over the Western North Pacific Based on GPS Radio Occultation, Island Soundings, and Numerical Models

**DOI:** 10.3390/s19010155

**Published:** 2019-01-04

**Authors:** Fang-Ching Chien, Jing-Shan Hong, Ying-Hwa Kuo

**Affiliations:** 1 Department of Earth Sciences, National Taiwan Normal University, No. 88, Section 4, Ting-Chou Road, Taipei 11677, Taiwan; 2 Central Weather Bureau, Taipei 10048, Taiwan; rfs14@cwb.gov.tw; 3 UCAR Community Programs, University Corporation for Atmospheric Research, Boulder, CO 80301, USA; kuo@ucar.edu

**Keywords:** marine boundary layer height, GPS radio occultation, FORMOSAT-3/COSMIC

## Abstract

This paper estimates marine boundary layer height (MBLH) over the western North Pacific (WNP) based on Global Positioning System Radio Occultation (GPS-RO) profiles from the Formosa Satellite Mission 3 (FORMOSAT-3)/Constellation Observing System for Meteorology, Ionosphere, and Climate (COSMIC) satellites, island soundings, and numerical models. The seasonally-averaged MBLHs computed from nine years (2007–2015) of GPS-RO data are inter-compared with those obtained from sounding observations at 15 island stations and from the European Centre for Medium-Range Weather Forecasts (ECMWF) Reanalysis (ERA-Interim) and National Centers for Environmental Prediction Global Forecast System (NCEP GFS) data over the WNP from 2012 to 2015. It is found that the MBLH using nine years of GPS-RO data is smoother and more consistent with that obtained from sounding observations than is the MBLH using four years of GPS-RO data in a previous study. In winter, higher MBLHs are found around the subtropical latitudes and over oceans east of Japan, which are approximately located within the paths of the North Equatorial Current and the Kuroshio Current. The MBLH is also significantly higher in winter than in summer over the WNP. The above MBLH pattern is generally similar to those obtained from the analysis data of the ERA-Interim and NCEP GFS, but the heights are about 200 m higher. The verification with soundings suggests that the ERA-Interim has a better MBLH estimation than the NCEP GFS. Thus, the MBLH distributions obtained from both the nine-year GPS-RO and the ERA-Interim data can represent well the climatological MBLH over the WNP, but the heights should be adjusted about 30 m lower for the former and ~200 m higher for the latter. A positive correlation between the MBLH and the instability of the lower atmosphere exists over large near-shore areas of the WNP, where cold air can move over warm oceans from the land in winter, resulting in an increase in lower-atmospheric instability and providing favorable conditions for convection to yield a higher MBLH. During summer, the lower-atmospheric instability becomes smaller and the MBLH is thus lower over near-shore oceans.

## 1. Introduction

The Earth’s radiation budget in climate models is greatly determined by the radiative effect of stratocumulus clouds over oceans [1,2]. The tops of these clouds are closely related to the marine boundary layer height (MBLH). Obtaining a good estimate of the MBLH is thus very important for estimating the global energy budget in climate models. It is equally important in regional models as well, because the MBLH decisively determines the oceanic boundary layer processes involved in the model. Unfortunately, this task is not easy, owing to the fact that there are very few in situ observations over oceans. Since April 2006, when the Formosa Satellite Mission 3 (FORMOSAT-3)/Constellation Observing System for Meteorology, Ionosphere, and Climate (COSMIC) was launched, Global Positioning System Radio Occultation (GPS-RO) data with high resolution in the lower atmosphere have been successfully used in detecting the MBLH [3,4,5,6,7,8,9]. The advantage of using GPS-RO data for this task is that the GPS signal can pass through clouds and precipitation basically unaffected. Other advantages include the high vertical resolution (~100 m) of the GPS-RO profile, such that it can adequately identify the planetary boundary layer (PBL) top, and the excellent global coverage of GPS-RO data [10].

As a significant change in temperature and water vapor usually occurs near the PBL top, the PBL height (or MBLH) can be simply defined at an altitude where the greatest decrease is found in the vertical profile of bending angle, refractivity, temperature, or water vapor pressure. Chien et al. (hereafter, CHK16) estimated the MBLH over the western North Pacific (WNP) based on four years (2012–2015) of GPS-RO data from the FORMOSAT-3/COSMIC satellites [11]. They evaluated four methods of automatically detecting the MBLH against nearby observations from island radiosonde stations. The first method defines the MBLH by searching for the altitude of a maximum *P*,

(1) $$P\equiv \Delta \alpha (\frac{\Delta \alpha }{\Delta z}),$$

where Δ*α* is the bending angle lapse and Δ*α*/Δ*z* is the vertical gradient of bending angle [4]. This method considers both the maximal lapse rate and the maximal gradient of bending angle. The second method determines the MBLH by finding the break point in a refractivity profile [3,6,9]. The third method uses the altitude of the minimum gradient of bending angle to define the MBLH [3,5,7,8,9]. The final method is similar to the third one, but uses refractivity rather than bending angle. Among these four methods, CHK16 concluded that the first and the third methods that utilize bending angle to define the MBLH produce significantly better results than the other two, which use refractivity to estimate the MBLH. They also found that the first method (so-called MXP-BA: maximum P using bending angle) performs slightly better than the third one. By using the MXP-BA method to estimate the MBLH, CHK16 documented that the MBLH over the WNP is considerably higher in the winter than in the summer. A reason given by the authors to explain this result was that winter has a larger temperature difference between the ocean surface and the lower atmosphere than summer.

Even though four years (2012–2015) of GPS-RO data seemed to serve well in estimating the MBLH in CHK16, we found that the amount of data at several grid points over the WNP was less sufficient than originally thought, especially when subdivided into seasons. We thus decided to re-examine the MBLH in this paper by extending the analysis period of GPS-RO data from 2012–2015 to 2007–2015. In addition, we developed a scheme to automatically detect the PBL height based on the vertical profiles of potential temperature and water vapor mixing ratio from sounding observations. With this scheme, the MBLH was analyzed using sounding data from fifteen island stations over the WNP. The results could serve as a ‘ground truth’ for comparison with the MBLH from the GPS-RO and the model data. We also examined the correlation between the MBLH and sea surface temperature (SST)/lower-atmospheric instability, which was only briefly mentioned in CHK16 without in-depth investigation. Lastly, the MBLH from the GPS-RO data was inter-compared with those calculated from the numerical weather models.

## 2. Methods

CHK16 concluded that the MXP-BA method was the best choice in terms of estimating the MBLH from GPS-RO data. As a follow-up study, this paper applies the same method to analyze the MBLH over the WNP, while extending the analysis period back to the earlier dates of the FORMOSAT-3/COSMIC satellites. As the GPS-RO data do not cover the entirety of 2006, we chose to skip some data from this year and utilize nine years of GPS-RO data from December 2006 (the first month of the 2007 winter) to November 2015 (the last month of the 2015 autumn).

**Figure 1:**
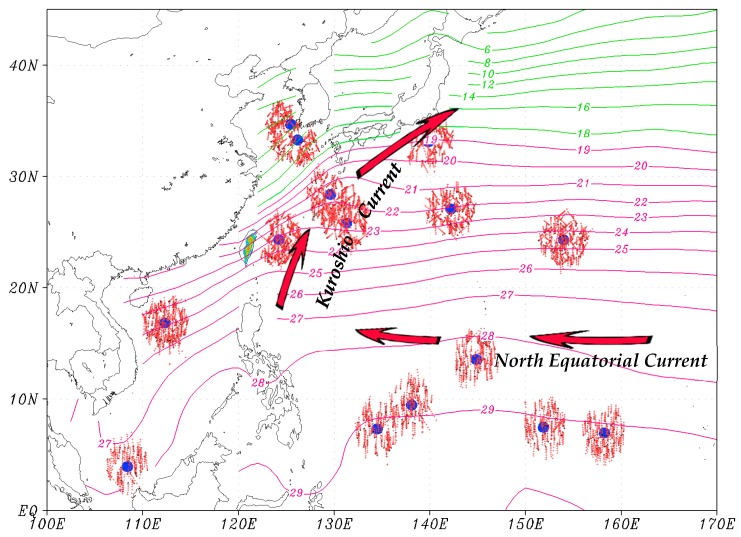
Locations of the 15 island sounding stations (blue dots) over the western North Pacific Ocean. Tiny red crosses show 0–5 km traces of the nearby GPS-RO profiles that are used for evaluation, with a black cross indicating the location at 5 km. The winter sea surface temperature (SST) averaged in December, January, and February, from 2012–2015, is plotted in green lines (2 °C interval) and purple lines (1 °C interval), using the National Oceanic and Atmospheric Administration (NOAA) Extended Reconstructed Sea Surface Temperature (ERSST) data. The Kuroshio Current and the North Equatorial Current are also denoted.

In CHK16, the MBLH over the WNP was estimated by the aforementioned methods using the GPS-RO profiles that are collocated with the fifteen island radiosonde stations (Figure 1) within a 250 km horizontal distance and 3 h time window. The MBLHs were then evaluated against those defined subjectively from the collocated twelve-hourly sounding observations (the mean vertical resolution of the sounding observations ranges from 160–190 m in the lower atmosphere at five out of the 15 island stations and varies from 300–390 m at the other stations). In total, there are 1411 of such cases available over the WNP in 2012–2015. Their traces passing through the lower atmosphere (≤5 km) are shown in Figure 1 for reference. However, it was very time-consuming to manually identify the MBLH of such cases. Therefore, in order to calculate the average MBLH using all available sounding observations over the WNP in 2012–2015, we developed a scheme in this paper to automatically detect the PBL height based on the vertical profiles of potential temperature and water vapor mixing ratio from sounding observations. First, the vertical gradient of potential temperature of a sounding below a 4 km altitude is checked. If the gradient at any level is larger than 0.003 K m^−1^ and 1.5 times larger than the gradient at the level below, this particular level is chosen as a potential PBL top because the stability there significantly increases. For a single sounding observation, there could be many levels satisfying these criteria. The vertical gradient of moisture at each of these levels is then computed by subtracting the height-weighted average water vapor mixing ratio, extending 500 m above from that extending 500 m below. The PBL top is defined at the level where the moisture gradient is the largest among all potential levels. If none of the above criteria are satisfied, the PBL height is registered as an undefined value.

The MBLH from the GPS-RO data was further compared with those calculated from the analysis data of numerical weather models, including the European Centre for Medium-Range Weather Forecasts (ECMWF) Reanalysis (ERA-Interim) and the National Centers for Environmental Prediction Global Forecast System (NCEP GFS). The analysis data (0 h) of these two global models were collected twice daily from 2012 to 2015. The horizontal resolutions are both 0.5° × 0.5°, but the vertical resolutions are slightly different. The former has 16 levels below 500 hPa, with a 50 hPa interval from 500 to 750 hPa and a 25 hPa interval from 750 to 1000 hPa, while the latter has 13 levels below 500 hPa, with a 50 hPa interval from 500 to 900 hPa and a 25 hPa interval from 900 to 1000 hPa. We first extracted water vapor pressure (*P_w_*), dry air pressure (*P*), and temperature (*T*) from the data sets, and then calculated RO refractivity (*N*) using the following equation:

(2) $$N=77.6\frac{P}{T}+3.73\times 10^{5}\frac{P_{w}}{T^{2}}.$$

By setting the model refractive index *n* = 1 + 10^−6^ *N*, the bending angle *α* as a function of the impact parameter *a* can be obtained from the Abel transform,

(3) $$\alpha (a)=-2a\int_{a}^{\infty }\frac{d(\ln n)/dx}{\sqrt{x^{2}-a^{2}}}dx,$$

where *x* = *nr* and *r* is the radius of a point on the raypath from a local curvature center [12]. Finally, the MBLH at each grid point can be estimated using the bending angle with the aforementioned MXP-BA method.

## 3. MBLH from Island Soundings

**Figure 2:**
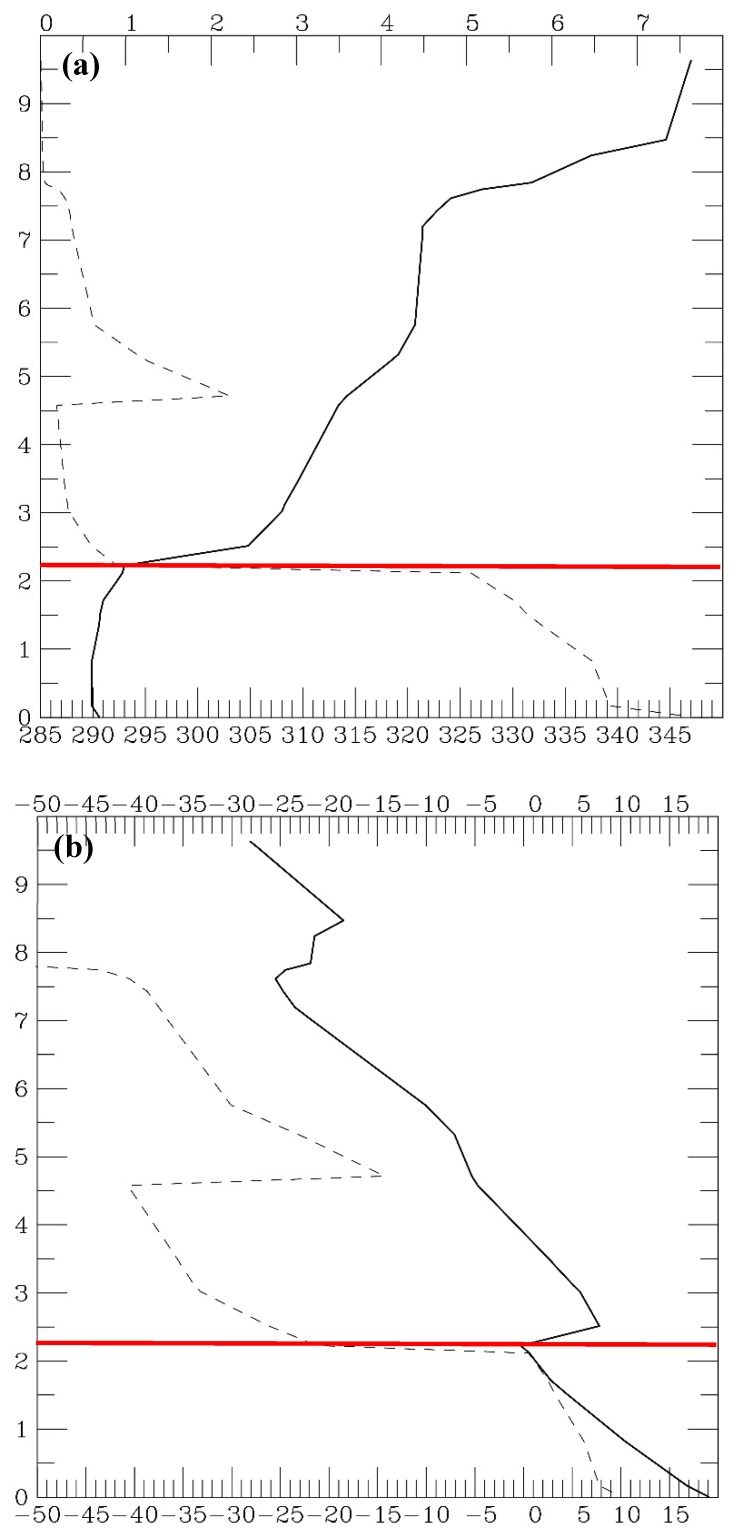
An example of an island sounding observation taken at Minamitorishima (24.3° N, 154° E) at 00:00 UTC 17 March 2012. (**a**) Potential temperature (K, scale on the bottom) and water vapor mixing ratio (g·kg^−1^, scale on the top) are plotted in solid and dashed lines, respectively. (**b**) Temperature (°C) and dew point temperature are plotted in solid and dashed lines, respectively. The estimated MBLH is indicated by a red line. Height is from 0 to 10 km.

All the cases in CHK16 were tested using the aforementioned scheme of automatic PBL height detection based on the sounding observation, and we found that the heights were close to those identified subjectively in CHK16. Figure 2 shows an example of such sounding observations taken at an island station (Minamitorishima) at 00:00 UTC 17 March 2012. This is the same case as presented in CHK16, except that potential temperature and water vapor mixing ratio are plotted, instead of bending angle and refractivity from a collocated GPS-RO profile. The MBLH of this case is objectively detected by the scheme at 2297 m, where the vertical gradient of potential temperature is large and moisture sharply decreases upward, and is very close to that identified in CHK16.

**Figure 3:**
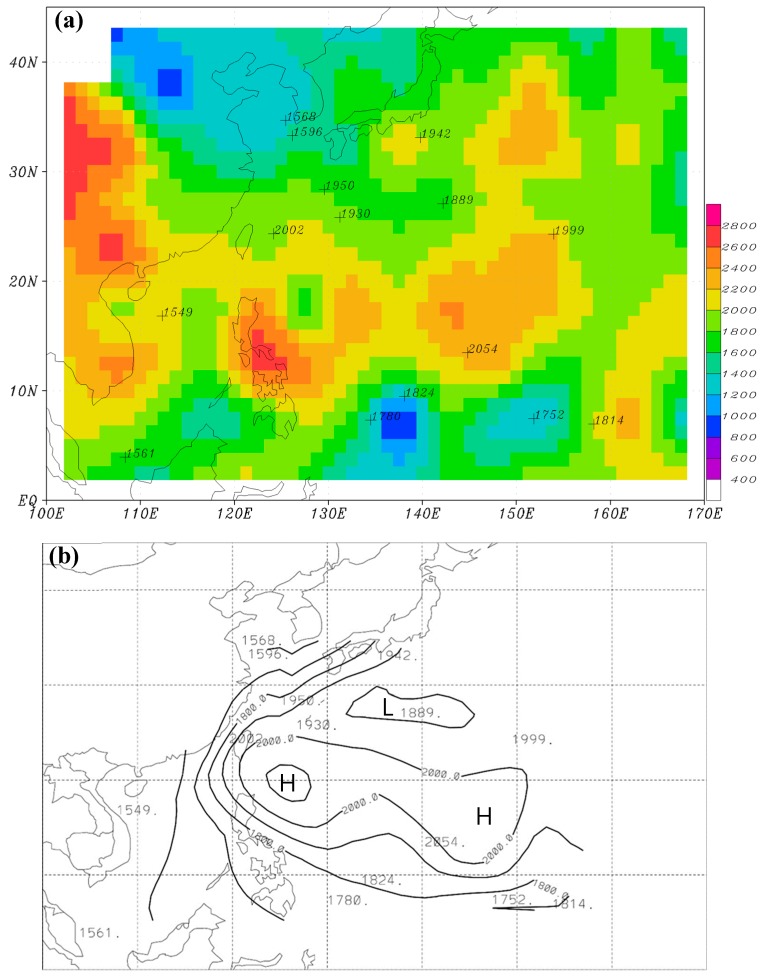
(**a**) The seasonally-averaged MBLH for winter using GPS-RO data in 2012–2015 (shading), with numerals showing the MBLH computed from island soundings. Cross points denote the locations of soundings. (**b**) Numerals are MBLH from island soundings, same as in (**a**). Contour lines are plotted with a 100 m interval using the C1 surface interpolation method.

We applied this scheme to all sounding observations at the 15 island stations over the WNP from December 2011 to November 2015. Their radiosonde station identifications are 47169, 47185, 47678, 47909, 47918, 47945, 47971, 47991, 59981, 91212, 91408, 91413, 91334, 91348, and 96147. The seasonally averaged MBLHs at all stations (hereafter, H_IS_) are calculated for winter and shown in numerals in Figure 3a, along with those computed from the 2012–2015 GPS-RO data (color shading, hereafter H_RO_). This H_RO_ is the same as in CHK16. In order to obtain a horizontal distribution of H_IS_ using the limited data from the 15 stations over the WNP, we further applied the C1 surface interpolation method [13] to plot the contour lines for H_IS_ (Figure 3b). Figure 3b shows high H_IS_ over the subtropical regions of the WNP, slightly lower values over oceans south of Japan, and low H_IS_ over the Yellow Sea and the South China Sea. Compared with H_RO_ (Figure 3a), H_IS_ has a similar pattern overall except that the local maximum at 120–130° E is placed a little northward.

**Figure 4:**
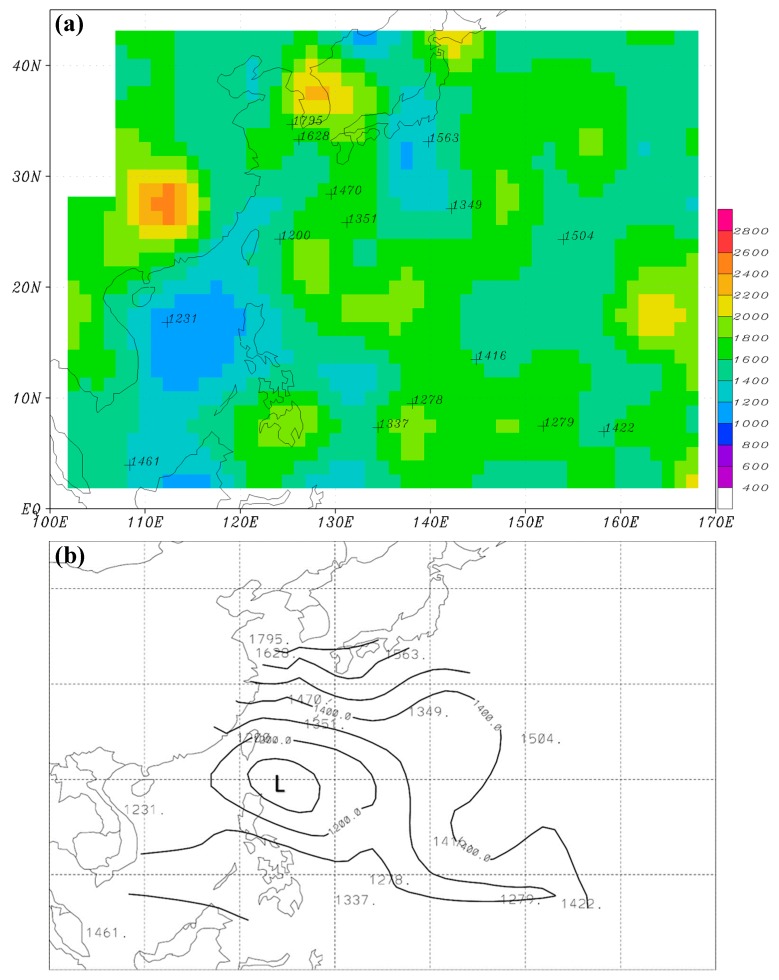
(**a**) The seasonally-averaged MBLH for summer using GPS-RO data in 2012–2015 (shading), with numerals showing the MBLH computed from island soundings. Cross points denote the locations of soundings. (**b**) Numerals are MBLH from island soundings, same as in (**a**). Contour lines are plotted with a 100 m interval using the C1 surface interpolation method.

The average MBLHs in summer (Figure 4) are significantly lower than those in winter (Figure 3) over the WNP, regardless of whether they are judged by the analysis of GPS-RO data (H_RO_) or from that of island soundings (H_IS_). In summer, however, the horizontal distributions of H_RO_ (Figure 4a) and H_IS_ (Figure 4b) are not as well-matched as in winter. The lower MBLHs are located around 120–130° E in Figure 4b, but around 110–120° E and a little southward in Figure 4a. Because spring and autumn are transitional seasons, their MBLHs fall between those of winter and summer; to be terse, their figures are not shown.

As stated in CHK16, the MBLHs from the widespread GPS-RO data were grouped into 5° × 5° boxes and the seasonally-averaged MBLH for each box was computed over the WNP, as shown in Figure 3a and Figure 4a for winter and summer, respectively. We applied interpolation to these MBLHs to obtain H_RO_ at the 15 island locations, and compared them with those of H_IS_. Table 1 shows that the correlation coefficient (hereafter, CC) between H_RO_ of the four-year data and H_IS_ at the 15 stations is positive in winter (0.37), but negative in summer (−0.05). In spring and autumn, the CCs are also positive at 0.42 and 0.29, respectively. Considering both spatial and seasonal relations together, the CC is 0.41 overall (ALL). The average difference between H_RO_ and H_IS_ for the 15 stations is about −40 m in winter, suggesting that MBLHs estimated from GPS-RO data are on average slightly lower than those from island soundings. In summer, however, the reverse is true and the average difference is larger. During the transitional seasons of spring and autumn the average differences are also positive. Grouping all seasons together, the overall difference (ALL) is positive (34 m). However, it is noted in Table 1 that the average absolute differences for all groups are around 220 m, meaning that H_RO_ and H_IS_ are not as close as the average difference may suggest.

## 4. MBLH from the Nine-Year GPS-RO Data

As discussed in the previous section, there are some similarities between H_RO_ and H_IS_, but large absolute differences still exist. It is suggested that the problem might be partly because of an insufficient quantity of data at some grid boxes using the GPS-RO data from 2012 to 2015. The quantity of island sounding data should not be a problem, however, because most stations have two soundings per day, resulting in 2920 soundings over four years (at most). Even for the case of seasonal averages, the amount is still around 700 soundings. We thus decided to re-calculate H_RO_ by extending the analysis period of the GPS-RO data from 2012–2015 to 2007–2015.

**Figure 5:**
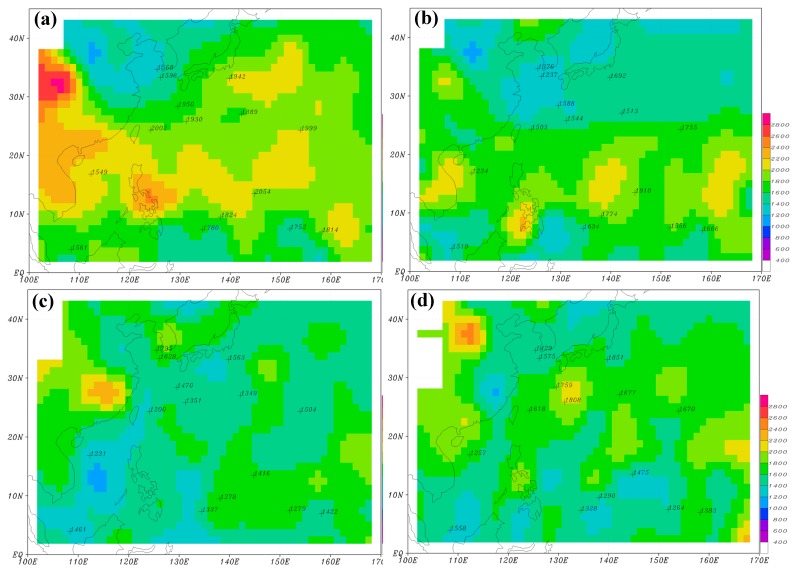
The seasonally-averaged MBLH for (**a**) winter, (**b**) spring, (**c**) summer, and (**d**) autumn, using the GPS-RO profiles from 2007 to 2015. The numerals denote the MBLH computed from island soundings.

Figure 5 shows seasonally-averaged H_RO_ using nine years of GPS-RO data from 2007 to 2015, along with H_IS_ displayed in numerals. In general, the horizontal distribution of H_RO_ over the WNP in winter (Figure 5a) has a similar pattern to that of the four-year GPS-RO data (Figure 3a). For example, higher H_RO_ (≥2000 m) are concentrated between 10° and 25° N extending from the northern South China Sea, through the Philippines, and farther to the east at 140°–155° E. Another area of higher H_RO_ is found over oceans east of Japan. These regions are roughly located within the paths of the North Equatorial Current and the Kuroshio Current, judged by the distribution of ocean currents [14] and mean sea surface temperature over the WNP (Figure 1). The major difference, however, is the magnitude of the MBLH. The local maxima and minima of H_RO_ obtained from the nine-year GPS-RO data become lower and higher, respectively, than those from the four-year data. This suggests that with more years of GPS-RO data used in the calculation, the MBLH becomes smoother and the result can be more representative of the climatological MBLH over the WNP. In summer, H_RO_’s over the WNP (Figure 5c) are about 1200–1800 m, which is significantly lower than those of winter (Figure 5a). The lowest H_RO_ (~1000 m) is found over the South China Sea. H_RO_’s in the spring and autumn (Figure 5b,d) fall between those of winter and summer. Winning et al. applied the method of minimum refractivity gradient (the fourth method mentioned in the introduction) to estimate the MBLH over the central North Pacific using GPS-RO data from 2007 to 2012 [15]. The left boundary of their studied domain (170° W) is close to our right boundary (170° E). They found higher MBLHs over the subtropical area and decreasing MBLHs towards the mid-latitude region near their left boundary (see their Figure 6), which is similar to what we found near the right boundary of Figure 5. In addition, the magnitudes of MBLH between these two figures are comparable. One major difference, however, is that the MBLH over the central North Pacific does not show the feature of higher values in winter than in summer, as we found over the WNP. This is of course closely related to the different features of ocean currents in these two regions.

**Figure 6:**
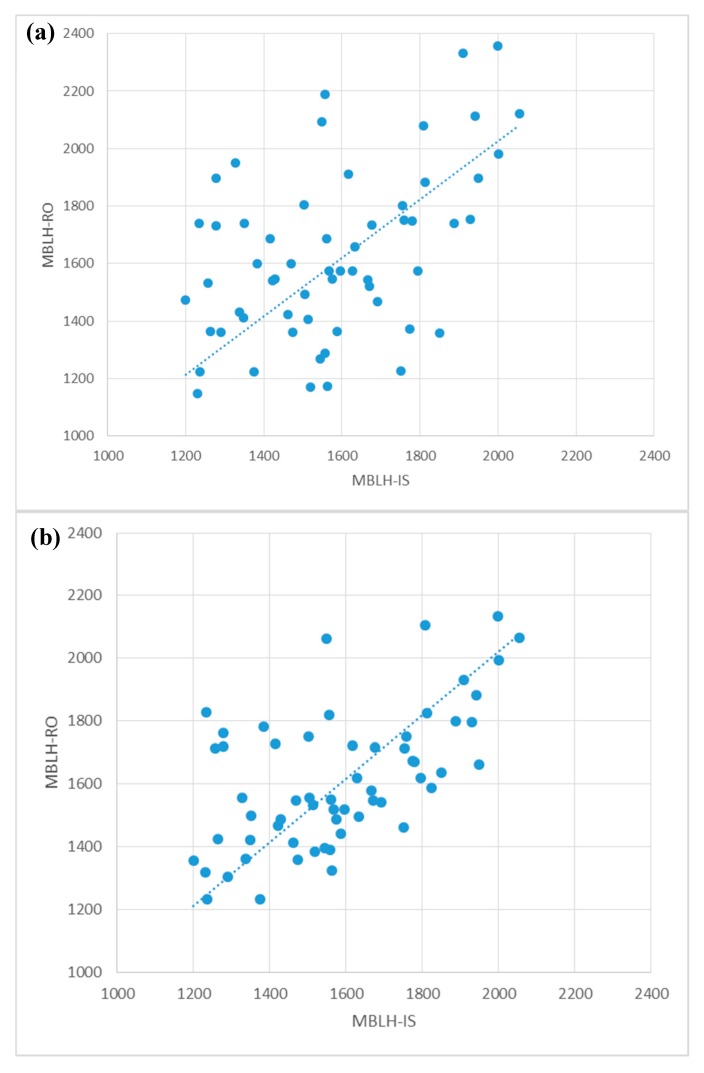
Scattered diagrams of the seasonally-averaged MBLH estimated from island radiosonde stations (the abscissa) and from GPS-RO profiles (the ordinate) using the GPS-RO data in (**a**) 2012–2015 and (**b**) 2007–2015. The sample size is 60 (15 island stations and 4 seasons).

Comparisons between Figure 5a,c and Figure 3a and Figure 4a show that H_RO_ of the nine-year GPS-RO data is more consistent with H_IS_ (in numerals) than H_RO_ of the four-year GPS-RO data. This can be more easily identified in the statistical analyses of Table 1, which presents higher CCs between H_RO_ of the nine-year GPS-RO data and H_IS_, than those between H_RO_ of the four-year GPS-RO data and H_IS_ for all seasons. Some of the CCs are even high enough to exceed the 95% confidence level. In addition, although the differences between H_RO_ and H_IS_ (H_RO_–H_IS_) are approximately the same, the absolute differences of H_RO_ for the nine-year GPS-RO data are much smaller than those of H_RO_ for the four-year GPS-RO data. The above results can be further illustrated in Figure 6, which displays scattered diagrams of seasonally-averaged H_RO_ and H_IS_ at the 15 island stations for all seasons (see the last row of Table 1 for the statistics). It is clear that although H_RO_ for the nine-year GPS-RO data and H_IS_ do not perfectly match (Figure 6b), their correlation is much better than that between H_RO_ for the four-year GPS-RO data and H_IS_ (Figure 6a). The inconsistency between H_RO_ and H_IS_ can result from many different error sources, including the imperfect algorithms that are used to automatically detect the MBLH, the interpolation processes that are needed to obtain H_RO_ at the island locations, and the different concepts and strategies that are applied in these two types of observational platforms. In addition, the different observational times can also contribute to this inconsistency, because while soundings are usually taken twice a day at 00:00 and 12:00 UTC, the GPS-RO data can be received at any time of day.

**Figure 7:**
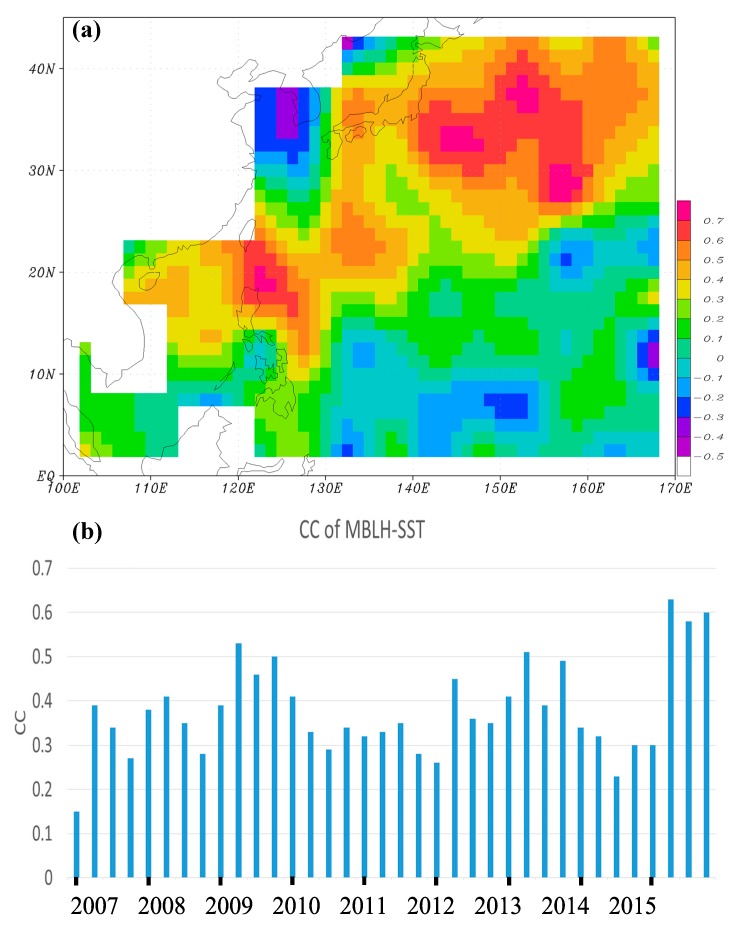
(**a**) Seasonal correlation coefficient (CC) between the seasonally-averaged MBLH and the lower-atmospheric instability from 2007 to 2015. (**b**) Spatial correlation coefficient between the seasonally-averaged MBLH and SST for the four seasons (started by winter) from 2007 to 2015.

As aforementioned, the relatively high MBLH regions are approximately located within the paths of the North Equatorial Current and the Kuroshio Current over the WNP. These warm ocean currents flow under relatively colder air, especially in the winter, providing favorable conditions for convection and higher MBLHs. In the summer, the temperature difference between the ocean surface and the atmosphere is much smaller, resulting in lower MBLHs. In this study, we further examined the CC between the seasonally-averaged H_RO_ and SST from 2007 to 2015, finding that the coefficients are mostly negative over the WNP (figure not shown). This is because SST is usually higher in summer than in winter, but the MBLH is reversed over the WNP. However, when SST is changed to the lower-atmospheric instability, which is defined as the difference between SST and the atmospheric temperature at 925 hPa, the CC becomes positive over large areas of the WNP extending northeastward from the South China Sea to oceans east of Japan (Figure 7a). These regions are relatively closer to the land. In winter, when cold air moves over warm oceans from the land, the lower-atmospheric instability increases, favoring the development of convection and a higher MBLH. One exception, however, is near the Yellow Sea region, where a cold ocean current is present in the winter (Figure 1). Far away from the land, the correlation between the MBLH and the instability is weak towards the central North Pacific. Although the seasonal correlation between the seasonally-averaged H_RO_ and SST is not positive, their spatial CCs over the WNP are all positive for each season from 2007 to 2015 (Figure 7b). In other words, the MBLH tends to be higher over oceans where SST is higher for the same season, although the CCs are not very high (around 0.3 to 0.5).

## 5. MBLH from Numerical Models

**Figure 8:**
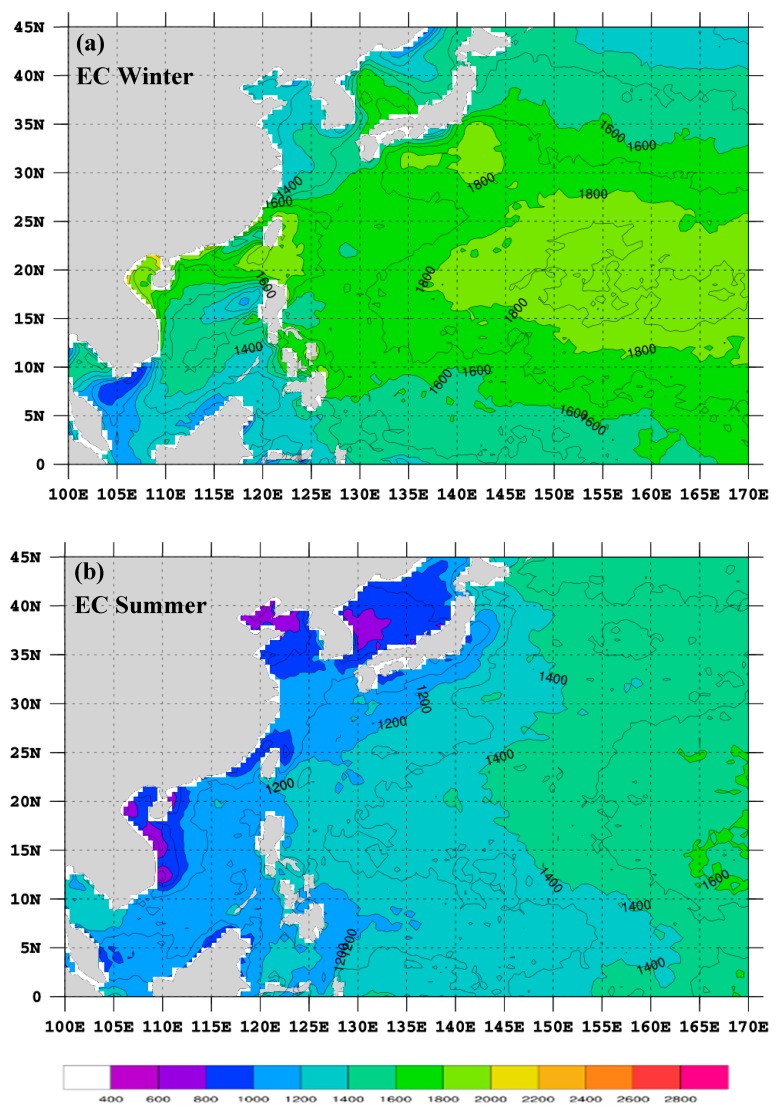
The seasonally-averaged MBLH (shown in both color and contour) for (**a**) winter and (**b**) summer using the ERA-Interim data (EC) from 2012 to 2015 based on the MXP-BA method. The scale is denoted at the bottom and the contour interval is 100 m. Grid points over land are masked.

We further computed the MBLH over the WNP based on the analysis data of ECMWF ERA-Interim (hereafter, EC) and the NCEP GFS using the method introduced in Section 2. Figure 8 presents the seasonally-averaged MBLH (hereafter, H_EC_) for winter and summer using the EC data from 2012 to 2015. The winter H_EC_ shows the highest values over a subtropical area (approximately between 10° N and 25° N) to the east of 140° E (Figure 8a). The height is roughly higher than 1800 m, but lower than 2000 m. H_EC_ also slightly exceeds 1800 m in small regions to the southeast of both Japan and Taiwan. Other than these areas, H_EC_ is all lower than 1800 m. Compared with the winter H_RO_ of the nine-year GPS-RO data (Figure 5a), H_EC_ has, to some extent, a similar higher-MBLH pattern over the aforementioned regions, but the height is about 200 m lower. Even so, some differences still exist between H_EC_ and H_RO_. For example, MBLH is large over the South China Sea and oceans to the east of the Philippines in H_RO_, but this is not observed in H_EC_.

In summer, H_EC_ exhibits a clear pattern of smaller values near the Asia continent and increasing eastward away from the continent (Figure 8b). The lowest MBLH is below 1000 m over near-shore oceans, and the highest MBLH is about 1400–1600 m toward the central North Pacific. The summer H_RO_ (Figure 5c) generally has a similar pattern to H_EC_, with lower values near the coastal areas and higher values toward the central North Pacific, except that the gradients are smaller. The small gradients are partly attributed to the fact that H_RO_ does not display MBLH as low as that of H_EC_ near the coastal areas. This is caused by the coarse resolution of the GPS-RO data, because H_RO_ is obtained by taking an average of all available observations inside a 5° × 5° box, while H_EC_ has a high horizontal resolution of 0.5° × 0.5°.

**Figure 9:**
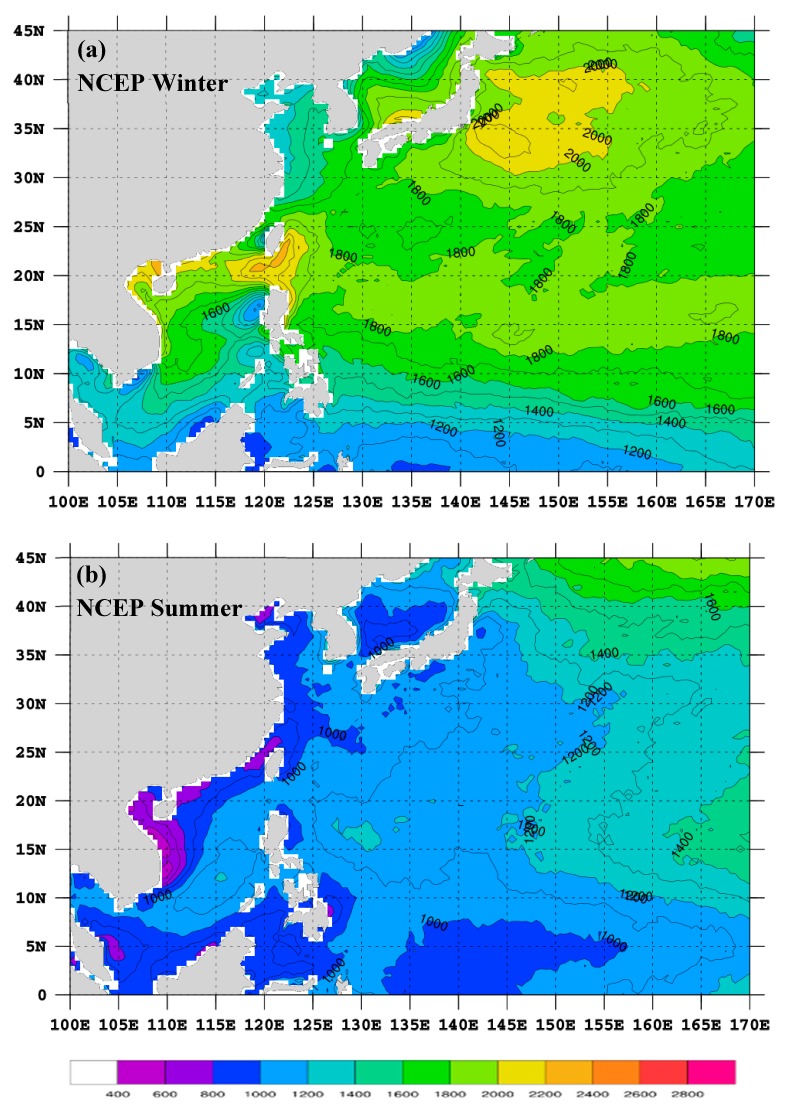
The seasonally-averaged MBLH (shown in both color and contour) for (**a**) winter and (**b**) summer using the NCEP GFS data (NC) from 2012 to 2015 based on the MXP-BA method. The scale is denoted at the bottom and the contour interval is 100 m. Grid points over land are masked.

The seasonally-averaged MBLH (hereafter, H_NC_) using the NCEP GFS data is presented in Figure 9. The winter H_NC_ shows a long belt (roughly 15–20° N) of high MBLH extending from oceans near Taiwan eastward to the central North Pacific (Figure 9a). Along this belt, H_NC_ is quite high near southern Taiwan (≥2000 m) and is about 1800–2000 m over other areas. High H_NC_ (≥2000 m) is also found over a broad ocean region to the east of Japan. Compared with H_EC_ (Figure 8a), this pattern of H_NC_ is more similar to that of H_RO_ (Figure 5a). However, H_NC_ is still about 200 m lower than H_RO_, except over oceans east of Japan. The summer H_NC_ (Figure 9b) is generally similar to that of H_EC_ (Figure 8b), but the MBLH is about 200 m lower over most parts of oceans.

Table 2 shows that the CC between H_EC_ and H_IS_ at the 15 stations is positive and large in winter (0.86), but negative in summer (−0.30). In spring and autumn, the CCs are also quite large at 0.79 and 0.73, respectively. Considering both spatial and seasonal relations together, the CC is 0.77 overall (ALL). Except for summer, all the CCs exceed the 95% confidence level. The mean differences between H_EC_ and H_IS_ are around −200 m in the four seasons and ALL, and their mean absolute differences are around 200 m. These results suggest that H_EC_ is highly correlated with H_IS_ in the horizontal pattern over the WNP, except for summer, but the MBLH is overall underestimated (~185 m lower) compared with that from the island soundings. The CCs between H_NC_ and H_IS_ are generally similar to those between H_EC_ and H_IS_, except that CC in summer is positive (0.38). However, the mean differences between H_NC_ and H_IS_ are generally larger. Therefore, H_NC_ is also highly correlated with H_IS_, but the MBLH is on average about 212 m lower than that from the island soundings.

## 6. Discussion and Conclusions

This paper estimates the MBLH over the WNP using GPS-RO profiles from the FORMOSAT-3/COSMIC satellites, island sounding observations, and analysis data of the ERA-Interim and the NCEP GFS. The MXP-BA method was chosen to analyze the MBLH from the GPS-RO data from 2007 to 2015 based on the previous study (CHK16). The MBLHs were inter-compared with those obtained from radiosonde observations at the 15 island stations and from the numerical model data over the WNP from 2012 to 2015. Potential temperature and water vapor mixing ratio profiles of the sounding observations were used to automatically detect the MBLH using a scheme developed in this study. In addition, the correlation between the MBLH and sea surface temperature (SST)/lower-atmospheric instability over the WNP was examined in this paper.

In general, the seasonally-averaged MBLH using nine years of GPS-RO data has a similar pattern to that of the four-year GPS-RO data (as presented in CHK16) over the WNP. For example, in winter, higher MBLHs are found around the subtropical latitudes and over oceans east of Japan, which are approximately located within the paths of the North Equatorial Current and the Kuroshio Current. The MBLH is significantly higher in winter than in summer over the WNP. However, the local maxima and minima of MBLH become lower and higher, respectively, when more GPS-RO data are used in the calculation. Comparisons show that the MBLH of the nine-year GPS-RO data is more consistent with that obtained from sounding observations at the 15 island stations when compared with the MBLH using four years of GPS-RO data. These results suggest that with more years of GPS-RO data included in the analysis, the MBLH becomes smoother and the result can be more representative of the climatological MBLH over the WNP.

The MBLHs from the analysis data of the EC and NCEP GFS generally have a similar pattern to those calculated from the nine years of GPS-RO data in either winter or summer, but the height is slightly underestimated (about 50–200 m lower). When compared with those from island soundings, the MBLHs from the models are also around 200 m lower overall (~185 m for EC and ~212 for NCEP), but their MBLH patterns are highly correlated over the WNP. The EC has a slightly better MBLH estimation over the WNP than the NCEP GFS because the overall difference is smaller. In addition, the high-resolution model data help present more clearly an MBLH pattern of lower heights near the continent and higher heights toward the central North Pacific than do the GPS-RO data in summer. A similar pattern can also be found in the summer MBLH of the island soundings (Figure 4b). The GPS-RO data are based on real, although not in situ, observations, while the EC and NCEP data are based on numerical analyses. The shortcoming of the former is the limited amount and scarce distribution of the data, which resulted in MBLH differences when compared with those from the models. The EC and NCEP data are able to provide MBLH in a rather high resolution over the WNP. However, the heights may contain errors because they were obtained through several procedures that could introduce uncertainties during computation, not to mention the uncertainty of numerical analyses over the wide-open ocean. The island soundings are relatively true in situ observations compared with the GPS-RO, but they are extremely scarce in space. Nonetheless, they can be used for verification and comparison. A true answer of what the MBLH over the WNP should look like may require more in situ observations, which are not easy to come by. However, from the inter-comparisons of this study, we can conclude that the MBLH distributions obtained from both the nine-year GPS-RO data and the EC data can represent well the climatological MBLH over the WNP, but the heights should be adjusted about 30 m lower for the former and ~200 m higher for the latter. The real MBLH should be in between the two. If a high resolution MBLH pattern is required, the EC results can be chosen. Otherwise, the GPS-RO results would be a good choice.

Lastly, a positive correlation between the MBLH and the instability of the lower atmosphere exists over large near-shore areas of the WNP, where cold air can move over warm oceans from the land in winter, resulting in an increase in lower-atmospheric instability. This consequently provides favorable conditions for convection and yields a higher MBLH in winter. During summer, the lower-atmospheric instability becomes smaller and the MBLH is thus lower over near-shore oceans. The MBLH increases eastward away from the continent over the WNP. The positive CC becomes smaller over the subtropical oceans toward the central North Pacific because the lower-atmospheric instability does not change much there from winter to summer. The higher MBLH around this region in winter than in summer is related to the path of the warm North Equatorial Current, which can be more dominant in winter than in summer.

## Figures and Tables

**Table 1:** Correlation coefficient (CC) and mean difference between the seasonally-averaged MBLH estimated from GPS-RO profiles (H_RO_) and that from island radiosonde stations (H_IS_). The mean absolute difference is also shown behind the slash. Double asterisks denote a confidence level of 95%, and a single asterisk for 90%. H_RO_ has two groups, one using four years of GPS-RO data in 2012–2015 (left column) and the other using nine years of GPS-RO data in 2007–2015 (right column). ALL is putting all seasons together in the calculation. DJF—December, January, February; MAM—March, April, May; JJA—June, July, August; SON—September, October, November.

Seasons/Sample Number	4 Years		9 Years	
	CC	H_RO_-H_IS_	CC	H_RO_-H_IS_
DJF(winter)/15	0.37	−39.4/218.3	0.55 **	−45.4/134.9
MAM(spring)/15	0.42	4.7/253.0	0.45 *	3.1/149.3
JJA(summer)/15	−0.05	108.1/213.9	0.19	95.2/157.8
SON(fall)/15	0.29	64.1/205.9	0.50 **	69.1/165.2
ALL/60	0.41 **	34.4/222.8	0.58 **	30.5/151.8

**Table 2:** Same as Table 1, except for correlation coefficient (CC) and mean difference between H_EC_ and H_IS_ (left column), and between H_NC_ and H_IS_ (right column).

Seasons/Sample Number	EC		NCEP	
	CC	H_EC_-H_IS_	CC	H_NC_-H_IS_
DJF(winter)/15	0.86 **	−188.3/188.3	0.74 **	−104.6/136.8
MAM(spring)/15	0.79 **	−187.6/192.6	0.88 **	−218.4/218.4
JJA(summer)/15	−0.30	−228.1/228.1	0.38	−358.0/358.0
SON(fall)/15	0.73 **	−139.7/148.3	0.76 **	−168.3/176.9
ALL/60	0.77 **	−185.9/189.3	0.84 **	−212.3/222.5
